# Strategies for Alzheimer’s Disease: The Role of Exercise and Omega‐3 Fatty Acids in Attenuating Neuroinflammation and Disease Progression

**DOI:** 10.1002/alz70861_109001

**Published:** 2025-12-23

**Authors:** Zachary L. Morcos, Deepesh Khanna

**Affiliations:** ^1^ KPCOM, Nova Southeastern University, Davie, FL USA; ^2^ KPCOM, Nova Southeastern University, Clearwater, FL USA

## Abstract

**Background:**

Alzheimer’s disease (AD) is a neurodegenerative disorder currently affecting approximately 34 to 40 million people worldwide and 6.9 million Americans. AD is characterized by significant neuroinflammation that results in progressive cognitive decline. Currently available pharmacologic therapies only offer limited benefits. As a result, there is increased interest in non‐pharmacological interventions. This narrative review examines the effects of aerobic, anaerobic, and high‐intensity interval training (HIIT) exercise modalities and omega‐3 (O3) supplementation on AD progression, prevention, and symptom relief.

**Method:**

A narrative literature review was conducted following PRISMA guidelines. Searches were performed in PubMed and Google Scholar using the following keywords: *“Alzheimer’s disease” AND (“aerobic exercise” OR “anaerobic training” OR “interval training”) AND (“omega‐3” OR “DHA” OR “EPA” OR “fish oil” OR “polyunsaturated fatty acids” OR “PUFA”)*. The search was limited to original research articles published in peer‐reviewed journals and written in English. Studies were eligible if they evaluated neuroinflammatory outcomes related to both exercise and omega‐3 interventions in the context of Alzheimer’s disease. Key outcomes of interest included amyloid‐β levels, pro‐inflammatory cytokines, and NF‐κB activation. Studies not meeting these inclusion criteria were excluded. Relevant data were extracted and qualitatively synthesized for thematic analysis.

**Result:**

All exercise modalities demonstrated reductions in amyloid‐β (Aβ) burden, modulation of inflammatory cytokines, and preservation of cognitive function across various species studied. O3 supplementation shows promise in enhancing neurogenesis and reducing inflammation, particularly in the early stages of cognitive impairment; however, it does not appear to significantly affect moderate or late‐stage cognitive decline.

**Conclusion:**

Future research should focus on standardizing intervention methods and investigating combined strategies in larger, long‐term clinical trials. Integrating lifestyle‐based interventions represents a plausible, holistic approach to managing and preventing AD and similar neurodegenerative diseases.

